# Interventional pulmonology for patients with central airway obstruction: An 8-year institutional experience

**DOI:** 10.1097/MD.0000000000005612

**Published:** 2017-01-13

**Authors:** Chia-Hung Chen, Biing-Ru Wu, Wen-Chien Cheng, Chih-Yu Chen, Wei-Chun Chen, Te-Chun Hsia, Wei-Chih Liao, Chih-Yen Tu, Wu-Huei Hsu

**Affiliations:** a Division of Pulmonary and Critical Care Medicine, Department of Internal Medicine, China Medical University Hospital; b Department of Respiratory Therapy; c Graduate Institute of Clinical Medical Science; d Department of Internal Medicine, Hyperbaric Oxygen Therapy Center; e School of Medicine; f Department of Life Science, National Chung Hsing University, Taichung, Taiwan.

**Keywords:** central airway obstruction, electrocautery, flexible bronchoscopy, self-expandable metallic stents, ultraflex

## Abstract

Patients with central airway obstruction (CAO) may need endobronchial intervention to relieve their symptoms. This report is on a single-center experience of using interventional bronchoscopy in terms of complications and survival. This retrospective study was conducted in a university hospital and involved 614 patients (464 men, 150 women; mean age, 60.2 years) with benign (n = 133) and malignant (n = 481) tracheobronchial disease who received 756 endobronchial intervention procedure during the period 2008 to 2015. Survival was analyzed using the Kaplan–Meier method, while the log-rank test was used for comparisons. A total of 583 patients (95%) achieved endoscopic success after interventional bronchoscopy. Four (0.7%) died within 24 hours of the procedure, while the major morbidities were halitosis (n = 41, 6.7%) and iatrogenic pneumonia (n = 24, 3.9%). Repeat procedures due to recurrent airway obstruction were done on 45 patients with benign conditions and on 60 with malignancies. The median survival after the procedure in patients with lung cancer, other metastatic cancer, and esophageal cancer was 166, 228, and 86 days, respectively. Between patients with inoperable lung cancer and CAO after therapeutic bronchoscopy and patients without CAO, there was no statistically significant difference in survival (*P* = 0.101). Interventional bronchoscopy is a safe and effective procedure that may be recommended for CAO. Patients with lung metastases have similar lengths of survival as patients with primary lung cancer. Patients with advanced lung cancer and CAO have similar survival as those without CAO.

## 1 Introduction

Central airway obstruction (CAO) can result from a variety of disease processes and cause significant morbidity and mortality. The most common etiology of malignant CAO is lung cancer, with an estimated 20% to 30% of patients developing complications associated with airway obstruction.^[1]^ The most common causes of nonmalignant CAO are postintubation and posttracheostomy tracheal stenosis, with an incidence of 10% to 22%.^[2]^ Regardless of malignant or nonmalignant etiology, the signs and symptoms of CAO are often extremely disturbing and life-threatening. Malignant CAO is especially associated with a major reduction in quality of life and poor prognosis.^[3]^

Successful bronchoscopic intervention restores the patency of the central airways and provides symptomatic and functional improvement. Therapeutic flexible bronchoscopy (e.g., laser therapy, electrocautery, and argon plasma coagulation) and insertion of self-expandable metallic stents (SEMS) have become effective palliative treatment for patients with CAO due to malignant or nonmalignant disease.^[4–9]^ Covered SEMS have also been used to seal tracheoesophageal fistulas and bronchial dehiscence after lung transplantation.^[10–11]^

This study aimed to analyze the baseline characteristics, clinical features, overall symptomatic response, complication rate, and overall survival of patients with CAO who received therapeutic bronchoscopy in an 8-year period. Aside from comparing CAO patients with and without malignant etiologies, this study also compared the overall survival of patients with inoperable lung cancer and CAO who received therapeutic bronchoscopy and chemotherapy and those treated with chemotherapy alone.

## 2 Materials and methods

### 2.1 Enrolled patients

From January 2008 to December 2015, 614 patients (mean age ± standard deviation [SD], 60.2 ± 14.9 years; range, 18–87 years) underwent interventional bronchoscopy at China Medical University Hospital, a university-affiliated hospital in Taiwan. Informed consent was obtained from each patient and/or their family before the procedure. Hospital records and procedure notes were reviewed in order to extract the following: age, sex, type of underlying tracheobronchial disease, bronchoscopic appearance (presence of intraluminal disease, extrinsic compression, or airway laceration), performance of bronchoscopic procedure, stenting, and occurrence of procedure-related complications. The main outcome measure was overall survival in all recruited patients. The secondary outcome measures included the immediate results of endoscopy.

A clinical result was defined as an improvement in dyspnea or a resolution of hemoptysis, depending on the indication. Endoscopic efficacy was defined as restoration >50% of tracheal or bronchial caliber 24 hours after the procedure. Improvement in atelectasis was defined as the re-expansion of at least 1 initially collapsed lobe at 24 hours. Complications in the first 48 hours were classified as hemorrhagic, obstructive, or mortality.

The hospital's Internal Review Board approved the study (DMR98-IRB-335) and waived the requirement for informed consent.

### 2.2 Bronchoscopic procedure

Patients underwent the procedure under conscious sedation with intravenous midazolam (5 mg) and a local anesthetic with 2% xylocaine solution. An Olympus PSD-60 unipolar electrode endobronchial electrocautery with therapeutic bronchoscope (BF-1T260; Olympus; Tokyo, Japan) was applied when the obstruction was caused by malignant exophytic tracheobronchial lesions. Ultraflex SEMS (Boston Scientific; Natick, MA) were used alone or after endobronchial electrosurgery, and the SEMS was implanted under flexible bronchoscopic rather than fluoroscopic guidance. The length of the airway stenosis and type of stent (with or without cover) were evaluated by flexible bronchoscopy and chest computed tomography (CT), if a CT scan was available before the stent implant. If stent repositioning was required, a biopsy forceps (FB-15C-1, Olympus; Tokyo, Japan) was used to hold the end of the stent while a thread was filed through the last loop of the meshwork. By smoothly pulling or pushing the thread, the stent position was adjusted. This entire procedure was performed in 1 session without fluoroscopic guidance.

Balloon dilatation was performed based on the physician's decision. The balloon was inflated to 6 to 8 of the standard atmosphere and maintained for 15 seconds. If the oxyhemoglobin saturation was ≤90% or if the patient could not endure hypoxia, the procedure was immediately stopped and the balloon was deflated and removed. Inflation was performed 3 to 5 times to obtain the desired results. All of the patients were monitored by pulse oximeter and electrocardiogram during the procedure.

### 2.3 Assessment of stent condition

A follow-up bronchoscopy was performed 48 hours after stent placement. The presence of incomplete stent expansion or an incomplete stented airway lumen was recorded so that postprocedure factors could be evaluated in follow-up bronchoscopic studies. In addition, each patient underwent bronchoscopic examination 1 week after implantation and every 3 to 6 months thereafter to evaluate stent position and degradation, granulation tissue formation, and airway alignment. If new or progressive symptoms including dyspnea, severe cough, increased mucous production, or other symptoms that suggested stent fracture occurred, additional bronchoscopy was performed.

### 2.4 Definition of interventional bronchology and SEMS complications

Chest X-ray was arranged after the days of procedure to evaluate if any complication occurred after procedure. Iatrogenic pneumonia due to procedure was defined as new onset of pulmonary infiltration from chest X-ray and also accompanied with symptoms such as fever, cough, increased mucous production or dyspnea after procedure. All possible complications related to SEMS placement were confirmed with bronchoscopic examination. According to patients’ records, complications included stent migration, granulation tissue formation, stent fracture, and pneumothorax. SEMS fracture was defined as physical breakage.

### 2.5 Statistical analysis

The data were compiled and analyzed using the statistical software SPSS for Windows, version 17.0 (Chicago, IL). All continuous variables were reported as mean ± SD, while categorical variables were reported as numbers and percentages. Differences in categorical variables were examined using Fisher exact test. Statistical significance was set at a 2-sided *P* < 0.05. Survival was calculated from the date of therapeutic bronchoscopy. The study end was set at December 31, 2015. Survival time was estimated using the Kaplan–Meier method. Log-rank test was performed to compare the results between groups.

## 3 Results

### 3.1 Patients

From January 2008 to December 2015, 614 patients (mean age, 60.2 ± 14.9 years; range, 18–87 years) with benign (n = 133) and malignant (n = 481) tracheobronchial disease received 756 endobronchial intervention procedures. The indications for interventional bronchoscopy were listed in Table 1. The female/male ratio was 0.32.

**Table 1:**
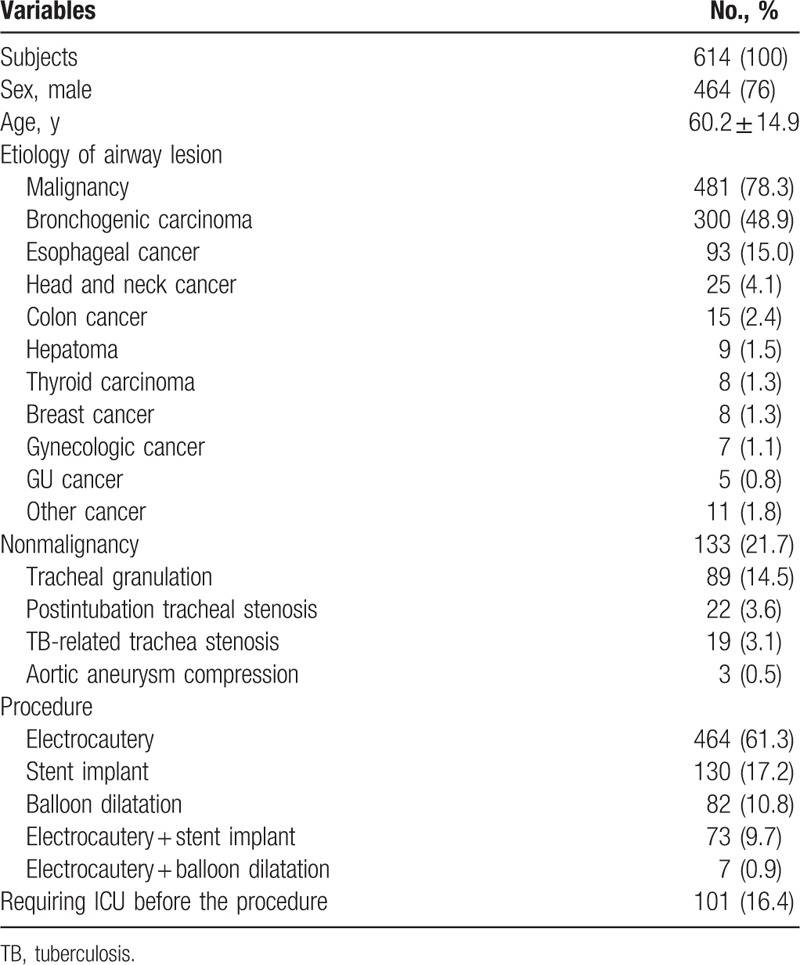
Baseline characteristics of patients with CAO (n = 614) and endobronchial interventions (n = 756).

Malignant disease caused the central airway lesions in 481 patients (78%), and the most common etiologies were lung cancer (n = 300, 62.3%) and esophageal cancer (n = 93, 19.3%). Among patients with malignant central airway disorders, 127 (26.4%) were treated with stent alone, 281 (58.4%) by electrocautery alone, and 73 (15.2%) by both stent and electrocautery. Electrocautery were performed 2 times in 42 patients, 3 times in 15, and 4 times in 3.

Benign disease contributed to central airway lesions in 133 patients (22%), and the most common etiologies were tracheal granulation (n = 89, 66.9%) and postintubation tracheal stenosis (n = 22, 16.8%). Among patients with benign central airway disorders, 37 (28.2%) were treated with balloon dilatation alone, 86 (65.6%) by electrocautery alone, and 7 (5.3%) by both balloon dilatation and electrocautery. Three (2.3%) patients with compressed trachea due to aortic aneurysm underwent SEMS replacement. Electrocautery were performed twice in 12 patients and thrice in 2. Balloon dilatation was performed 2 times in 20 patients, 3 times in 8 patients, and 4 times in 3. Moreover, 101 patients (16.4%) with respiratory failure required intensive care unit care before the interventional bronchoscopy.

**Figure 1:**
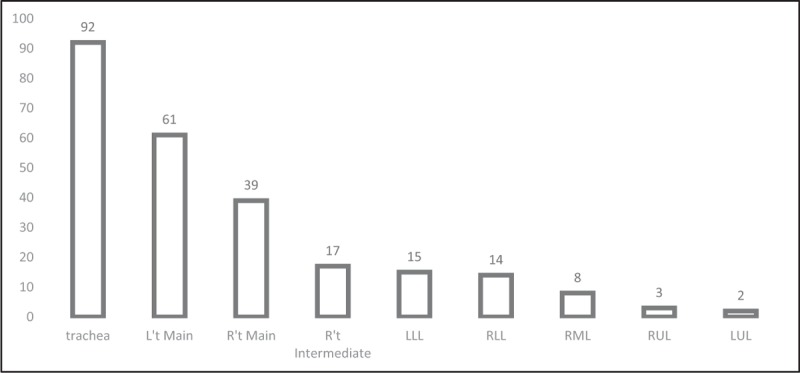
Location and number of stent implantations.

The locations and etiologies of CAO were summarized in Table 2. Tumor invasion (n = 420) and tumor compression (n = 130) were the most common indications for interventional bronchoscopy. Ten patients had 3 sites of tumor invasion, while 49 patients had 2 sites. The 203 patients with benign (n = 3) and malignant (n = 200) tracheobronchial disease received 251 Ultraflex SEMS (3 for benign and 248 for malignant conditions). The most common locations of stent implantation were the trachea (n = 92, 36.7%) and the left main bronchus (n = 61, 24.3%) (Fig. 1). In terms of stent size used (Table 3), the most common sizes were 14 × 30 mm^2^ (n = 64, 25.5%) and 14 × 40 mm^2^ (n = 50, 19.9%).

**Table 2:**
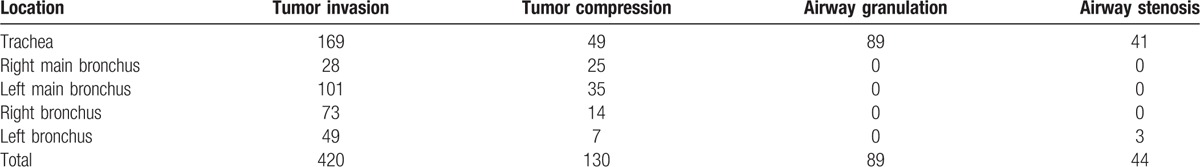
Locations and etiology of central airway lesions.

**Table 3:**
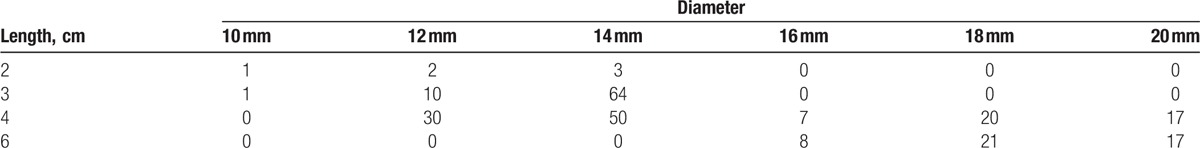
Summary of the size of tracheobronchial stents implanted in patients with CAO.

Based on the success, mortality, and morbidity rates after the interventional procedure for CAO (Table 4), 583 patients (95%) achieved endoscopic success. However, 4 patients (0.7%) with malignant CAO died within 24 hours of the procedure due to respiratory failure caused by massive bleeding. Major morbidities were usually noted after the stent implantation, the most common of which were halitosis (n = 41, 6.7%) and iatrogenic pneumonia (n = 24, 3.9%).

**Table 4:**
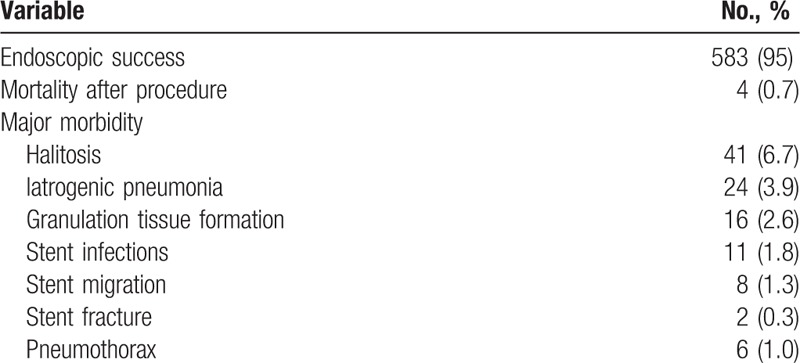
Success, mortality, and morbidity rates after interventional procedure with CAO.

**Figure 2:**
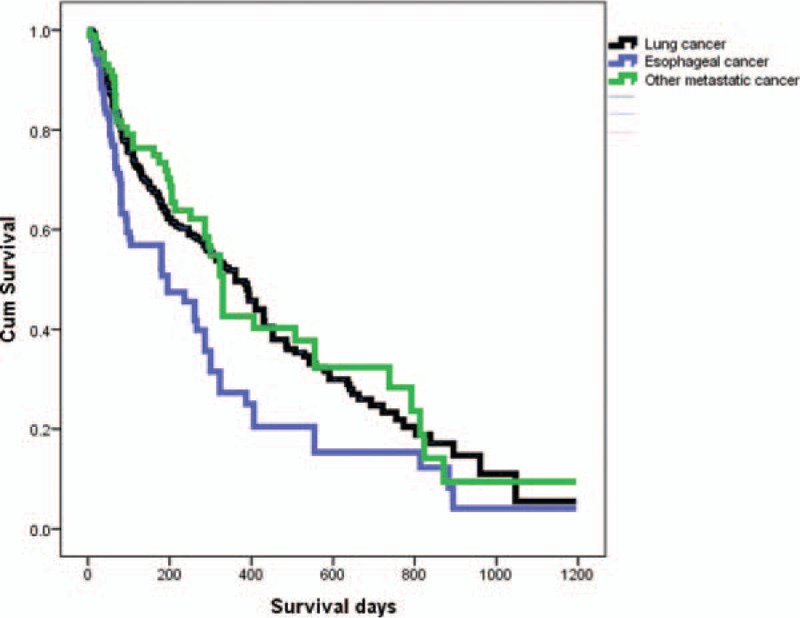
Overall survival rate after interventional bronchoscopy stratified by histologic type of malignant central airway obstruction.

The overall survival rates in the different etiologies of CAO were compared by Kaplan–Meier survival curves (Fig. 2). The etiologies of malignant CAO were further classified into lung cancer (n = 300, 62.4%), esophageal cancer (n = 93, 19.3%), and other metastatic cancers (n = 88, 18.3%). By the end of the study, 191 (63.7%) lung cancer, 64 (68.8%) esophageal cancer, and 47 (53.4%) metastatic cancer patients had died. Seven patients (3 with lung cancer, 3 with esophageal cancer, and 1 with other metastatic cancer) died within 2 weeks after the procedure. Median survival after interventional bronchoscopy was 166 days for the lung cancer group, 198 days for the other metastatic cancer group, and 86 days for the esophageal cancer group. Patients with esophageal cancer had a shorter life expectancy after interventional bronchoscopy compared to the other groups.

**Figure 3:**
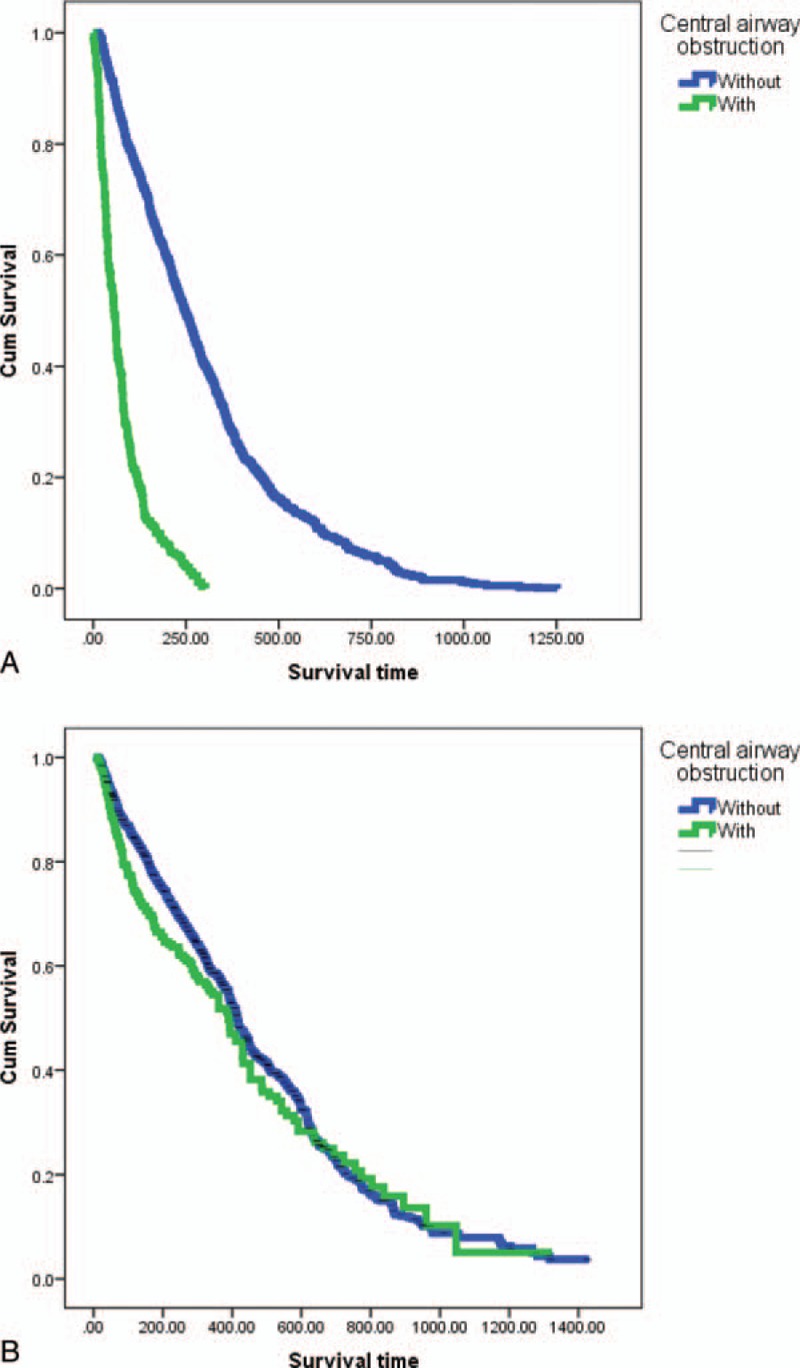
Kaplan–Meier survival curve of patients with and those without central airway obstruction treated with interventional bronchoscopy in the periods of (A) 2003 to 2007 and (B) 2008 to 2015.

To determine if therapeutic bronchoscopy really benefitted inoperable lung cancer, the patients were classified according to a different cohort. Patients with inoperable lung cancer who received chemotherapy alone, regardless of CAO (patients from January 2003 to December 2007) were compared to those who received therapeutic bronchoscopy and chemotherapy with CAO or chemotherapy alone if without CAO (patients from January 2008 to December 2015). Log-rank test showed that survival rate was significantly higher in patients with inoperable lung cancer but no CAO (n = 1000) than in patients with CAO (n = 262) (*P* < 0.001) (Fig. 3A). However, there was no statistically significant difference in the survival of patients with inoperable lung cancer and CAO (n = 273) after therapeutic bronchoscopy and patients without CAO (n = 1359) (*P* = 0.101) (Fig. 3B).

## 4 Discussion

Interventional bronchoscopy is generally considered safe and can provide immediate symptomatic relief of dyspnea, making it a worthwhile palliative therapy. In this study, 95% of patients achieved endoscopic success after interventional bronchoscopy, and only 4 (0.7%) died within 24 hours after procedure. Patients with advanced lung cancer and CAO treated with a combination of therapeutic bronchoscopy and chemotherapy had similar survival rates compared to those with advanced lung cancer but no CAO.

The study hospital does not have Argon Plasma Coagulation or Laser. Only electrocautery is used for tumor dissection. The principle of electrocautery involves the use of high-frequency electrical currents via probe to coagulate or dissect tumor tissue. It is a simple technique that has the ability to produce rapid palliation and immediate tumor debulking.^[12]^ Its side effects include hemorrhage, airway perforation, aspiration pneumonia after debulking due to pus from the obstructive pneumonia, and endobronchial fire. In this study, 544 electrocautery procedures were performed in 447 patients. After the procedure, only 4 (0.7%) suffered massive bleeding and 24 (4.2%) were diagnosed with aspiration pneumonia.

Airway stents are classified into silicone and metal stents. Silicone stents are implanted using a rigid bronchoscope under general anesthesia, while metal stents are safely and quickly implanted using a flexible bronchoscope. However, some complications such as obstructive granulation tissue, stenosis at the ends of the stent, migration of the stent, mucous plugging with/without infection, and stent fracture happened after using metallic stents.^[13–15]^

Because of serious complications associated with the use of metallic tracheal stents, the United States Food and Drug Administration has warned that SEMS implantation should be considered in patients with benign airway disorders only after thoroughly exploring all other treatment options, such as surgical procedures or placement of silicone stents. In this study, 3 patients with aortic aneurysm compression had implanted SEMS as treatment for high risk for surgery. All 3 suffered from granulation tissue formation after SEMS implantation. Hence, SEMS for benign CAO should be restricted to highly selected patients.

In the study hospital, benign CAO due to postintubation or tuberculosis (TB)-related tracheal stenosis is usually treated with balloon dilatation first. Thirty-one (75.6%) patients need more than 2 more balloon dilatation procedures after the first. Since the pathogenic mechanism of postintubation or TB-related tracheal stenosis are different, different treatments for each entity should been proposed. Open surgery has an important role in the treatment of complex and recurrent stenosis, whereby the stenotic segment is resected surgically by subsequent end-to-end anastomosis.^[16]^ In patients with complex stenosis who are not candidates for surgery, or in whom this option has failed, the use of silicone stents is recommended.^[17]^

Airway complications in esophageal cancer patients may be caused by extrinsic compression of the airways by the tumor or by direct tumor invasion into the airways. These complications can result in airway stenosis and tracheoesophageal fistula.^[18]^ Unfortunately, at the time of diagnosis, a high percentage of patients with esophageal cancer have an advanced stage of the disease and only palliative treatment is applicable. Endoscopic stenting has gained acceptance as the preferred palliative therapy for airway complications.^[19]^ But even with tracheal stent implantation, esophageal cancer with CAO still has the poorest prognosis compared to other groups. By definition, CAO due to metastatic disease is a widespread disease, and both pulmonologists and patients assume a poor prognosis. However, in this study, the survival of such patients is not worse when compared to other patients with CAO due to lung cancer. Thus, even patients with metastatic disease should be considered candidates for interventional endoscopy.

An important finding is that patients with advanced lung cancer with locally treated CAO combined with systemic chemotherapy behave similarly to patients without CAO and treated with chemotherapy alone. The combination of therapeutic bronchoscopy and chemotherapy should be actively considered in all patients with lung cancer and CAO. Moreover, patients with CAO are at risk for postobstructive pneumonia and/or atelectasis. Any acute infection is a contraindication for chemotherapy. Endobronchial treatment of CAO helps to relieve postobstructive pneumonia and allows patients to receive additional treatment.^[20]^ In this study, 74% of patients can benefit by receiving chemotherapy after the local treatment of endobronchial obstruction. As such, lung cancer patients with CAO should be carefully evaluated as they may also be eligible to receive chemotherapy aside from local treatment and this may have a positive impact on survival.

Certain limitations of our study deserve to be acknowledged. First, this was a retrospective study and single-institution design. The retrospective nature of our study is a source of recall bias and does not allow us to evaluate improvements in quality of life. Prospective, multicenter trials are ideal and recommended in the future. Second, we compared 2 different cohorts (2003–2007 and 2008–2015) for confirmation that lung cancer patients with CAO could be improved survive rate after therapeutic bronchoscopy. Despite different cohorts, the histology of lung cancer patients with CAO were due to squamous cell carcinoma (n = 214, 71.3%), small cell lung carcinoma (n = 54, 18%), and adenocarcinoma (n = 32, 10.7%). There were no more new chemotherapy drugs when we treated squamous cell carcinoma and small cell lung carcinoma form 2003 to 2015. Hence, we can still concluded that the survival improved in CAO patients were not due to chemotherapy effect but due to the treated with therapeutic bronchoscopy.

## 5 Conclusion

Interventional bronchoscopy is a safe and effective procedure that is recommended for symptomatic CAO due to its almost immediate effect. Despite being an invasive procedure, its rate of complications is low. Benign CAO may also recur after the procedure and may require silicone stent implantation and surgery. Patients with lung metastases from other malignancies have as long a survival time as patients with primary lung cancer and should therefore be offered treatment. Lastly, patients with advanced lung cancer and malignant CAO treated with a combination of therapeutic bronchoscopy and chemotherapy have the same survival rate as those with advanced lung cancer without CAO and treated with chemotherapy only. Therapeutic bronchoscopy should be offered to patients with lung cancer and CAO.
